# Prolyl carboxypeptidase in Agouti-related Peptide neurons modulates food intake and body weight

**DOI:** 10.1016/j.molmet.2018.02.003

**Published:** 2018-02-08

**Authors:** Giuseppe Bruschetta, Sungho Jin, Jung Dae Kim, Sabrina Diano

**Affiliations:** 1 Program in Integrative Cell Signaling and Neurobiology of Metabolism, Yale University School of Medicine, New Haven, CT, 06520, USA; 2 Department of Obstetrics, Gynecology, and Reproductive Sciences, Yale University School of Medicine, New Haven, CT, 06520, USA; 3 Department of Neuroscience, Yale University School of Medicine, New Haven, CT, 06520, USA; 4 Department of Comparative Medicine, Yale University School of Medicine, New Haven, CT, 06520, USA

**Keywords:** Prolyl carboxypeptidase, NPY/AgRP, α-MSH, Food intake, Energy metabolism

## Abstract

**Objective:**

Prolyl carboxypeptidase (PRCP) plays a role in the regulation of energy metabolism by inactivating hypothalamic α-melanocyte stimulating hormone (α-MSH) levels. Although detected in the arcuate nucleus, limited PRCP expression has been observed in the arcuate POMC neurons, and its site of action in regulating metabolism is still ill-defined.

**Methods:**

We performed immunostaining to assess the localization of PRCP in arcuate Neuropeptide Y/Agouti-related Peptide (NPY/AgRP) neurons. Hypothalamic explants were then used to assess the intracellular localization of PRCP and its release at the synaptic levels. Finally, we generated a mouse model to assess the role of PRCP in NPY/AgRP neurons of the arcuate nucleus in the regulation of metabolism.

**Results:**

Here we show that PRCP is expressed in NPY/AgRP-expressing neurons of the arcuate nucleus. In hypothalamic explants, stimulation by ghrelin increased PRCP concentration in the medium and decreased PRCP content in synaptic extract, suggesting that PRCP is released at the synaptic level. In support of this, hypothalamic explants from mice with selective deletion of PRCP in AgRP neurons (*Prcp*^*AgRPKO*^) showed reduced ghrelin-induced PRCP concentration in the medium compared to controls mice. Furthermore, male *Prcp*^*AgRPKO*^ mice had decreased body weight and fat mass compared to controls. However, this phenotype was sex-specific as female *Prcp*^*AgRPKO*^ mice show metabolic differences only when challenged by high fat diet feeding. The improved metabolism of *Prcp*^*AgRPKO*^ mice was associated with reduced food intake and increased energy expenditure, locomotor activity, and hypothalamic α-MSH levels. Administration of SHU9119, a potent melanocortin receptor antagonist, selectively in the PVN of *Prcp*^*AgRPKO*^ male mice increased food intake to a level similar to that of control mice.

**Conclusions:**

Altogether, our data indicate that PRCP is released at the synaptic levels and that PRCP in AgRP neurons contributes to the modulation of α-MSH degradation and related metabolic control in mice.

## Highlights

• PRCP is expressed in the arcuate NPY/AgRP neurons.
• PRCP is released in the synaptic space following ghrelin stimulation.
• Male mice with PRCP deletion in NPY/AgRP neurons show leaner phenotype with decreased food intake on standard chow diet.
• Female mice with PRCP deletion in NPY/AgRP neurons show leaner phenotype with decreased food intake only on high fat diet.
• Selective blockade of PVN melanocortin receptors increases feeding in male mice with PRCP deletion in NPY/AgRP neurons.

## 1 Introduction

The arcuate melanocortin system comprises neurons expressing either the anorexigenic pro-opiomelanocortin peptide (POMC) or the orexigenic Neuropeptide Y/Agouti-related peptide (NPY/AgRP). These neurons project to overlapping target areas, including the paraventricular nucleus of the hypothalamus, where AgRP antagonizes the effect of α-melanocyte stimulating hormone (α-MSH), a product of the POMC gene, on melanocortin receptors (MCRs)-expressing neurons [1]. Furthermore, NPY/AgRP neurons send GABAergic projections to POMC neurons, inhibiting POMC neuronal activity [2], [3] and providing an additional regulatory mechanism onto POMC neurons.

Alpha-MSH is generated by extensive posttranslational processes that involve several enzymes, such as the prohormone convertases 1 and 2 (PC1 and PC2), carboxypeptidase E (CPE) and peptidyl α-amidating monooxygenase (PAM). Considering the complex pathway of posttranslational modifications for the generation of α-MSH, it is therefore possible that alterations in any step of this process may affect energy metabolism. Indeed, impaired POMC processing has been reported to induce obesity in rodents [4] and humans [5]. For example, inactivation of carboxypeptidase E induced obesity which is associated with high circulating levels of prohormones [4]. In humans, the obesity phenotype reported is associated with mutations in the human prohormone convertase 1 gene [5] and increased levels of unprocessed POMC. Studies on the regulation of these enzymes have shown that leptin, glucocorticoids, and thyroid hormones regulate PC1 and PC2 *in vivo* and *in vitro* [6], [7], [8], [9].

Despite the anorectic function of α-MSH [10], [11], peripheral administration of α-MSH does not reduce food intake suggesting that α-MSH is rapidly degraded. We have previously reported that Prolyl carboxypeptidase (PRCP) is a key enzyme responsible for the degradation of α-MSH [12]. PRCP gene-trap hypomorph (*PRCP*^*gt/gt*^) mice are leaner and have reduced food intake and increased hypothalamic α-MSH levels [12].

The serine protease PRCP cleaves peptides with a penultimate proline residue at the C-terminal. PRCP is expressed in many tissues including kidney, lung, heart, liver, adipose, as well as the brain [13]. It has been shown that PRCP can be present extracellularly either as membrane-bound or as soluble form [14], [15], [16], indicating its extracellular release in response to stimuli. Within the hypothalamus, PRCP is expressed in several neuronal populations [12], [17]. Although detected in the arcuate nucleus, limited PRCP expression has been observed in the arcuate POMC neurons [12]. Food deprivation induced up-regulation of PRCP both at the mRNA and protein expression levels [17], an event mediated by ghrelin via ghrelin receptors [17]. Because ghrelin receptors are expressed in NPY/AgRP but not in POMC neurons [18], [19], [20] and ghrelin excites NPY/AgRP neurons, this study was carried out to elucidate whether PRCP in NPY/AgRP neurons may play a role in the regulation of metabolism.

## 2 Material and methods

### 2.1 Ethics and animal husbandry

All animal studies on male and female mice were approved by Yale University Institutional Animal Care and Use Committee.

Animals had a free access to either a standard chow diet (SD; Harlan Teklad no. 2018, 18% calories from fat) for up to 3 months of age. All animals were kept in temperature and humidity controlled rooms, in a 12/12 h light/dark cycle, with lights on from 7:00AM to 7:00PM. Food and water were provided ad libitum, unless otherwise stated. Fasted animals were food deprived overnight (16 h).

For high fat diet experiments, female mice were exposed to high fat diet (45% fat; Research diet, Rodent Chow #D12451) starting at 6 weeks of age.

### 2.2 PRCP immunostaining

Three-month-old male mice were anesthetized and transcardially perfused with 0.9% saline containing heparin (10 mg/l) followed by fixative (4% paraformaldehyde, 15% picric acid, 0.1% glutaraldehyde in 0.1 M phosphate buffer [PB]). Brains were collected and post-fixed overnight before coronal sections were taken at every 50 μm. Sections were washed and then transfer to 10 nM sodium citrate buffer (pH 8.5) preheated to 80 °C in water bath. The sections were kept in this solution for 30 min. After washing and blocking with 4% normal goat serum, sections were incubated with primary antibodies (goat anti-PRCP, 1:2000; Santa Cruz Biotechnology, Cat# sc-49272) and the chicken anti-GFP antibody (1:5000; Abcam, ab13970) overnight at room temperature (RT). After several washes with PB, sections were incubated in the secondary antibodies (donkey anti-goat Alexa-Fluor 594; 1:200 in PB; Vector Laboratories and goat anti-chicken Alexa- Fluor 488; 1:500 in PB; Life Technologies) for 2 h at room temperature. Sections were mounted on glass slide with vectashield (Vector lab) and analyzed with a confocal microscope.

### 2.3 PRCP measurements from hypothalamic explants

Male mice were sacrificed by decapitation and whole brain immediately removed and put into a cutting solution (2.5 mM KCl, 6 mM MgCl_2_, 1 mM CaCl_2_, 1.25 mM NaH_2_PO_4_, 26 mM NaHCO_3_, 10 mM Glucose, and 250 mM Sucrose). A 2 mm thick slice of mediobasal forebrain including the PVN and ARC was prepared using a vibratome, and the hypothalamus was cut from the rest of the brain. Hypothalami were treated separately in 96-well plate with aCSF (124 mM NaCl, 3 mM KCl, 2 mM CaCl_2_, 2 mM MgCl_2_, 1.23 mM NaH_2_PO_4_, 26 mM NaHCO_3_, and 10 mM Glucose), equilibrated with 95% O_2_ and 5% CO_2_ and incubated at 37 °C. After 1 h equilibration period, hypothalami were incubated for 45 min in aCSF (basal period, 8 mM Glucose) and then incubated for 45 min in either a basal glucose-aCSF (8 mM glucose), a high glucose-aCSF (15 mM glucose), or in a basal glucose-aCSF (8 mM glucose) with 100 nM ghrelin [21]. We selected 8 mM and 15 mM glucose as low and high glucose conditions for the *ex vivo* hypothalamic explants and we added ghrelin to the low glucose (8 mM glucose)-treated explant according to our and others published protocols [22], [23], [24]. Tissue viability was verified by exposure to 56 mM KCl for 45 min. At the end of each period, supernatants were collected and frozen immediately. PRCP concentration of supernatants was assessed by the use of a commercial PRCP ELISA kit (MyBioSource, Cat#MBS929190) according to the manufacture's protocol, normalized to their basal period, and expressed as percent change. Hypothalamic explants that showed less PRCP secretion in response to KCl compared to the period before KCl exposure were excluded from the analysis.

We performed a similar experiment (as described above) on hypothalamic explants with the only difference that KCl exposure was not performed after the treatments in order to measure PRCP content in the hypothalamic explants by western blot analysis. For this experiment, hypothalamic explants were prepared as described above except for the KCl treatment. Cytoplasmic and synaptic proteins from hypothalamic explants were prepared using Syn-PERTM Synaptic Protein Extraction Reagent (Thermo scientific, Cat#87793) [25]. Protein concentrations were measured using the BCA kit (Thermo scientific, Cat# 23228 and 1859078). Ten ug of proteins were resolved in 10% SDS-PAGE and transferred to PVDF membrane (Millipore, Cat# IPVH 15150). Membranes were blocked with 5% dry milk in TBS (50 mM Tris–HCl, pH 7.5, 150 mM NaCl) for 1 h and then followed by overnight incubation at 4 °C with primary antibodies such as anti-PRCP antibody (Santa Cruz Biotechnology, Cat# sc-49272) and anti-GluR2 antibody (Millipore, Cat#MAB397). After 3 times washing with TBST (TBS including 0.05% Tween 20), anti-goat IgG conjugated to horseradish peroxidase (Santa Cruz Biotechnology, Cat# sc-2768) for anti-PRCP antibody or anti-mouse IgG-HRP (Santa Cruz Biotechnology, Cat# sc-2005) for anti-GluR2 antibody was incubated for 1 h and developed by ECL kit (Thermo scientific, Cat# 32016). Membranes were stripped using stripping buffer (Thermo scientific, Cat# 21059) and reused to detect β-actin (Sigma, Cat# A5441). Anti-GluR2 antibody was used as a synaptic marker [25], [26]. One-way ANOVA followed by the Bonferroni's multiple comparison test was used for statistical analysis.

### 2.4 Generation of mice with selective deletion of PRCP in AgRP neurons

We used the cre/lox technology to generate mice in which PRCP was selectively ablated in AgRP neurons (*Prcp*^*AgRPKO*^). The construct was developed by the EUCOMM (European Mouse Model Cell Repository; see www.eucomm.org). PRCP floxed mice (*Prcp*^*fl/fl*^) were generated by the Yale Genome Editing Center. *Prcp*^*fl/fl*^ mice were crossed with AgRP-cre mice that express Cre recombinase under the control of AgRP promoter [27]. As control mice, *Prcp*^*+/+*^*-AgRP-cre* positive and *Prcp*^*fl/fl*^*-AgRP-cre* negative littermates were used. All mice studied were on a mixed background (129SvEv, FVB and C57BL/6J).

### 2.5 Genotyping

Genomic DNA was isolated from tails or yolk sacs by standard methods. PRCP mice were genotyped by polymerase chain reaction (PCR parameters: 42 cycles, 93 °C for 30 s, 56 °C for 1 min, and 72 °C for 5 min). Amplification of a wild-type (WT) allele generated a 3.6- kb product, and a 4.1-kb product in the case of a mutant allele using the following primers: PRCP2 FRT5: 5′-ccttcagggccctcagtca -3′, PRCP2 FRT3: 5′-cagccatatttaaacattgagat -3. AgRP-cre alleles were identified by PCR analysis for cre sequences using the following primers: M263, 5′- atggctaatcgccatcttccagca -3 ′; and M262, 5′-gccctggaagggatttttgaagca -3 ′ (PCR parameters: 35 cycles, 94 °C for 30 s, 55 °C for 45 s, and 72 °C for 55 s).

### 2.6 Western blot analysis for PRCP

Hypothalami or punches from the ARC (medial portion) and VMH were prepared as previously described [24]. Protein concentrations were measured using the BCA kit (Thermo scientific, Cat# 23228 and 1859078). Twenty ug of proteins were resolved in 10% SDS-PAGE and transferred to PVDF membrane (Millipore, Cat# IPVH 15150). Membranes were blocked with 5% dry milk in TBS (50 mM Tris–HCl, pH 7.5, 150 mM NaCl) for 1 h followed by overnight incubation at 4 °C with primary antibodies such as goat anti-PRCP antibody (Santa Cruz Biotechnology, Cat# sc-49272). After 3 times washing with TBST (TBS including 0.05% Tween 20), anti-goat IgG conjugated to horseradish peroxidase (Santa Cruz Biotechnology, Cat# sc-2768) for anti-PRCP antibody was incubated for 1 h and developed by ECL kit (Thermo scientific, Cat# 32016). Membranes were stripped using stripping buffer (Thermo scientific, Cat# 21059) and reused to detect β-actin (Sigma, Cat# A5441). One-way ANOVA followed by the Bonferroni's multiple comparison test was used for statistical analysis.

### 2.7 Metabolic chamber recordings

Twelve weeks old male and female mice were acclimated in metabolic chambers (TSE Systems, Germany) for 4 days before the start of the recordings. Mice were continuously recorded for 3 days with the following measurements taken every 30 min: water intake, food intake, ambulatory activity (in X and Z axes), and gas exchange (O_2_ and CO_2_) (using the TSE LabMaster system, Germany). vO_2_, vCO_2_, and energy expenditure were calculated according to the manufacturer's guidelines (PhenoMaster Software, TSE Systems). The respiratory exchange rate (RER) was estimated by calculating the ratio of vCO_2_/vO_2_. Values were adjusted by body weight to the power of 0.75 (kg−0.75) where mentioned. EE was analyzed either after normalization by body weight or using the ANCOVA analysis. In addition, food intake was determined continuously by integration of weighing sensors fixed at the top of the cage, from which the food containers have been suspended into the sealed cage environment. Body composition was measured *in vivo* by MRI (EchoMRI; Echo Medical Systems, Houston, TX).

### 2.8 Real-time PCR

Total RNA from hypothalamus, brown adipose tissue, and liver was extracted from *Prcp*^*AgRPKO*^ and control animals using Trizol solution (Invitrogen).

Uncoupling protein 1 (UCP1) and Deiodinase 2 (DIO2) mRNA levels in the brown adipose tissue, Phosphoenolpyruvate carboxykinase 1 (PCK1) and glucose-6-phosphatase (G-6-Pase) mRNA levels in the liver, and pro-hormone convertase 1 (PC1), and pro-hormone convertase 2 (PC2) in the hypothalamus were measured by real-time PCR. A High Capacity cDNA Reverse transcription Kit (Applied Biosystems) was used for the reverse transcription. Real-time PCR (LightCycler 480; Roche) was performed with diluted cDNAs in a 20-μl reaction volume in triplicates. Primers used for this study are as follows: cat. no. Mm 00494069_m1 for PCK1, cat. no. Mm 00839363_m1 for G-6-Pase, Mm 00515664_m1 for DIO2, and Mm 01244861_m1 for UCP1, cat. no. Mm 00479023_m1 for PC1, cat. no. Mm 00500981_m1 for PC2 Mm 02619580_g1 for β-actin (Applied Biosystems). The calculations of average Cp values, SDs, and resulting expression ratios for each target gene were based on the Roche LightCycler 480 software.

### 2.9 Measurements of circulating hormones

Serum from blood samples was obtained by centrifugation at 3,000 rpm for 15 min, and each circulating hormone was determined using a commercially available ELISA kit as follows: total ghrelin (Rat/Mouse Total Ghrelin ELISA kit, cat. no. EZRGRT-91K; Millipore), and active ghrelin (Rat/Mouse Active Ghrelin ELISA kit, cat. no. EZRGRA-90K; Millipore), insulin (Rat/Mouse Insulin ELISA kit, cat. no. EZRMI-13K; Millipore), glucagon (cat. no. TR0100; Sigma), and corticosterone (ab108821, Corticosterone ELISA kit, Abcam). All procedures were performed by following the manufacturer's protocol.

### 2.10 Glucose and insulin tolerance tests

Glucose tolerance test was performed in 16 h-fasted animals. After the level of blood glucose was determined, fasted animals were injected intraperitoneally with glucose (2 mg/kg; DeltaSelect) in 0.9% saline. Blood glucose levels were then monitored at 15, 30, 60, and 120 min from the injection.

Insulin tolerance test was performed with mice fed ad libitum. After determination of basal blood glucose levels, each animal received an intraperitoneal injection of insulin, 0.75 U/kg (Actrapid; Novo Nordisk). Blood glucose levels were then measured at 15, 30, 60, and 120 min after insulin injection.

### 2.11 Hypothalamic α-MSH and β-endorphin measurements

Dissected hypothalami from fed *Prcp*^*AgRPKO*^ and controls mice were homogenized on ice with acid-ethanol. The homogenates were incubated at 20 °C for 30 min and then centrifuged at 3,000×*g* for 30 min at 4 °C. The resulting supernatant after centrifugation was transfer into new tubes and lyophilized. The samples were then reconstituted in 200 μl of RIA buffer and assayed for α-MSH (cat# RK-043-01) and β-endorphin (cat #RK-022-06) by RIA using kits from Phoenix Pharmaceuticals (Burlingame, CA) following the manufacturer's protocol.

### 2.12 cfos immunostaining

After overnight fasting, mice were anesthetized and transcardially perfused with 0.9% saline with heparin followed by fixative (4% paraformaldehyde, 15% picric acid, 0.1% glutaraldehyde in PBS). Brains were collected, post fixed overnight, and coronal sections were taken at every 50 μm.

Sections were washed and incubated with the rabbit anti-cfos antibody (Santacruz, 1:2000), and the chicken anti-GFP antibody (Life Technologies Corporation, 1:5000) in PB containing 4% normal goat serum, 0.1% glycine, and 0.2% Triton X-100 for 24 h at room temperature. After several washes with PB, sections were incubated in the secondary antibodies (biotinylated goat anti-rabbit immunoglobulin G [IgG]; 1:250 in PB; Vector Laboratories and goat antichicken Alexa-fluor 488; 1:200 in PB; Life Technologies) for 2 h at room temperature, then rinsed in PB five times, 10 min each time. Sections were then mounted with VectaShield antifade (Vector Laboratories). Fluorescent images of five to seven brain sections were captured with confocal microscope and analyzed by imaging Software (Image J).

### 2.13 α-MSH immunostaining

Immunofluorescence staining was performed using anti-α-MSH antibody (ab5087; Millipore Sigma). Brains were sectioned with a vibratome (50 μm), and sections were incubated overnight in anti-α-MSH antibody (diluted 1:2000 in 0.1 mol/L sodium phosphate buffer) and then incubated in secondary antibody (category no. A11016, Alexa Fluor 594–coupled donkey anti-sheep, 1:200 dilution; Life Technologies) for 2 h. Sections were then cover slipped with VECTASHIELD (H-1000; Vector Laboratories) for microscopic examination.

### 2.14 SHU9119 infusion in the PVN

For this study, male mice were bilaterally cannulated in the PVN (coordinates, bregma: anterior-posterior, −0.7 mm; lateral, ±0.25 mm; and dorsal-ventral, −5.2 mm): 5 *Prcp*^*AgRPKO*^ male mice injected with vehicle (1 ìl saline/side); 5 male control mice injected with vehicle (1 ìl saline/side); 5 *Prcp*^*AgRPKO*^ male mice injected with SHU9119 (1.5 μg/1ìl/side), and 5 male control mice injected with SHU9119 (1.5 μg/1ìl/side). All injections (over 4 min) were performed at 9AM. Food intake was measured at 2, 4, 6, and 24 h. Significance of the drug effects at different time points was determined by 2-way ANOVA.

One week later, mice were re-injected with SHU9119 and sacrificed by transcardiac perfusion (as described above) 24 h later and cfos staining performed and analyzed (as described above) in the PVN.

### 2.15 Statistics

We used software packages to analyze the data (Matlab R2009a and PASW Statistics 18.0) and plot the figures. First, we tested the homogeneity of variance across the different experimental conditions using Levene's or Barlett's test. When the p value was greater than 0.05 in these tests, homogeneity was assumed, and a parametric analysis of variance test was used. The student's t test was used to compare two groups. One-, two-, or three-way ANOVA were used as the other tests unless stated otherwise. Multiple comparisons were performed as described below. For repeated measures, a mixed model ANOVA, with time as a “within-subject repeated-measures” factor and treatments/genotype as a “between subject” factor, were used. Significant effects were followed with Fisher's PLSD post-hoc test with Bonferroni's correction. When homogeneity was not assumed, the Kruskal–Wallis nonparametric ANOVA was used, and the Mann–Whitney U test was used to determine post-hoc significance of differences between groups. Fisher's exact test was used to find differences in the number of cells activated by ghrelin in the electrophysiology recordings. A value of P < 0.05 was considered statistically significant. All data are shown as mean ± SEM unless stated otherwise.

## 3 Results

### 3.1 PRCP expression in AgRP neurons

**Figure 1:**
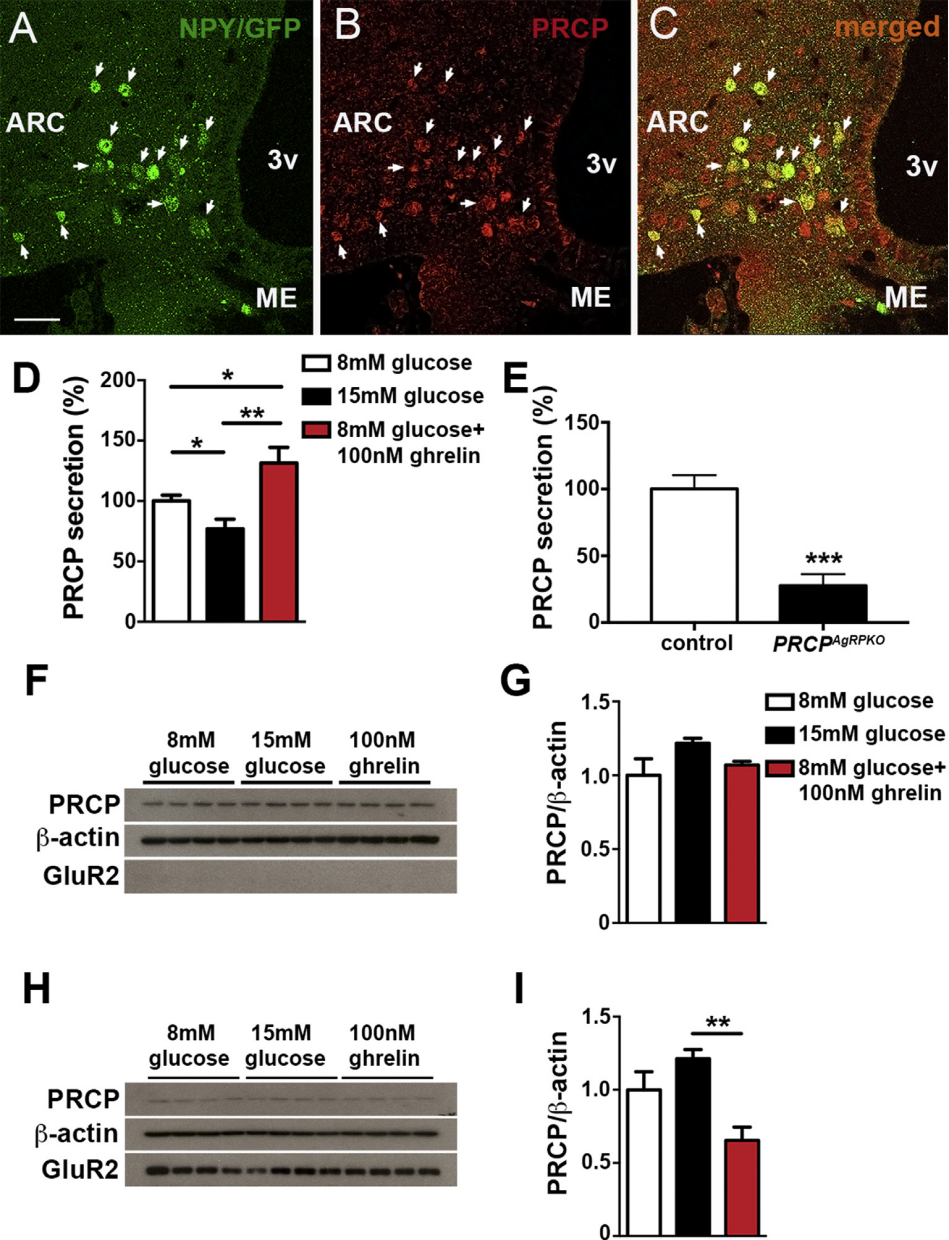
**PRCP expression in NPY/AgRP neurons and its secretion.** (**A**–**C**) Representative photographs of the hypothalamic arcuate nucleus showing GFP (NPY-GFP mouse; **A**) and PRCP immunolabeling (**B**). Analysis of double labeled cells (**C**) showed that 77.8 ± 2.39% of NPY neurons were positive for PRCP (arrowheads). (**D**) Graph showing the result of the PRCP measurements in hypothalamic explants of mice containing the PVN and the anterior arcuate nucleus. Ghrelin addition (100 nM) to the basal (8 mM glucose) aCSF induced a significant increase of PRCP in the medium compared to either basal aCSF or 15 mM glucose-containing aCSF (n = 7 per treatment). (**E**) Graph showing the result of the PRCP measurements in hypothalamic explants of *Prcp*^*AgRPKO*^ and control mice (n = 5 per group) containing the PVN and the anterior arcuate nucleus. Compared to controls, PRCP levels in the medium after ghrelin incubation were significantly lower in *Prcp*^*AgRPKO*^ mice. (**F** and **G**) Western blot images (**F**) and quantification of the density (**G**) of PRCP in cytoplasmic extracts from hypothalamic explants (n = 4 per group) incubated with either 8 mM Glucose, 15 mM Glucose or 8 mM Glucose plus 100 nM Ghrelin for PRCP, β-actin and GluR2. PRCP density was normalized to β-actin density. (**H** and **I**) Western blot images (**H**) and quantification of the density (**I**) of PRCP in synaptic extracts from hypothalamic explants (n = 4 per group) incubated with either 8 mM Glucose, 15 mM Glucose or 8 mM Glucose plus 100 nM Ghrelin for PRCP, β-actin and GluR2. PRCP density was normalized to β-actin density. 3v = third ventricle; ARC = arcuate nucleus; ME = median eminence. Bar scale in **A** (for all panels) represents 50 μm. Data represent the mean ± SEM.* = P < 0.05; ** = P < 0.01; *** = P < 0.001.

To assess whether PRCP is expressed in NPY/AgRP neurons, we performed immunostaining of PRCP in hypothalamic sections from NPY/GFP mice. The specificity of the antibody was tested using hypothalamic samples from whole body PRCP gene trap mice [12] (*Pcrp*^*gt/gt*^; Figure S1A). Our results showed that about 77.8 ± 2.39% of NPY neurons in the arcuate nucleus express PRCP (Figure 1A–C).

### 3.2 PRCP secretion in hypothalamic explants

PRCP can be released in response to stimulation, thus appearing in the extracellular medium or in biological fluids [15], [16], [28]. To assess whether PRCP is released after a stimulus shown to up-regulate PRCP expression levels, such as ghrelin [29], we performed a study in which PRCP levels were measured in the medium of *ex vivo* hypothalamic explants [22], [24]. Eight mM glucose plus 100 nM ghrelin induced a significant increase of PRCP in the medium compared to either 8 mM glucose-treated or 15 mM glucose-treated explants (n = 7 per group; Figure 1D). Compared to the explants treated with 8 mM glucose, 15 mM glucose-treated explants showed a significant lower PRCP levels in the medium (Figure 1D).

To assess the role of PRCP in AgRP neurons, we generated mice carrying a loxP *Prcp KO construct* (*Prcp*^*flox/flox*^, Figure S1B). Homozygous *Prcp*^*flox/flox*^ mice were then crossed with *AgRP*-Cre mice [27] to generate mice with selective PRCP deletion in AgRP neurons (*Prcp*^*AgRPKO*^) and their controls (*Prcp*^*flox/flox*^-*AgRP-cre* negative and *Prcp*^*+/+*^*-AgRP-cre*). To validate our model, we analyzed and found limited PRCP expression in the NPY/AgRP neurons of 3-months old male *Prcp*^*AgRPKO*^ (Figure S2A–C) compared to controls (Figure 1A–C) by immunohistochemistry. Furthermore, Western blot analysis of PRCP in punches of the medial region of the arcuate nucleus and the entire ventromedial nucleus (VMH) showed significant decrease in PRCP protein levels in the arcuate (1.00 ± 0.06 in controls versus 0.30 ± 0.07 in *Prcp*^*AgRPKO*^ mice; p = 0.002; Figure S2D) but not in the VMH (1.00 ± 0.11 in controls versus 0.78 ± 0.04 in *Prcp*^*AgRPKO*^ mice; p = 0.13; Figure S2E).

We then analyzed stimulated PRCP secretion from explants derived from male *Prcp*^*AgRPKO*^ mice compared to controls. PRCP levels in the medium of hypothalamic explants from *Prcp*^*AgRPKO*^ mice after ghrelin incubation were significantly lower than those observed in hypothalamic explants from control mice (Figure 1E).

### 3.3 PRCP expression in hypothalamic synaptic extracts

We then assessed PRCP expression levels in both cytoplasmic and synaptic extracts from hypothalamic explants of control mice. No differences in PRCP levels were found between groups in cytoplasmic extracts (Figure 1F, G). However, PRCP levels in synaptic extracts were significantly decreased in explants incubated in 8 mM glucose plus 100 nM ghrelin compared to either 8 mM alone or 15 mM glucose (Figure 1H, I), suggesting that ghrelin induces PRCP release from synaptic terminals. No significant differences were observed in PRCP levels of synaptic extracts from 8 mM to 15 mM exposed hypothalamic explants (Figure 1H, I). In all, these results, together with previous published data [15], [16], [28], suggest that PRCP is released by synaptic terminals.

### 3.4 Selective PRCP deletion in AgRP neurons alters energy metabolism in male mice

**Figure 2:**
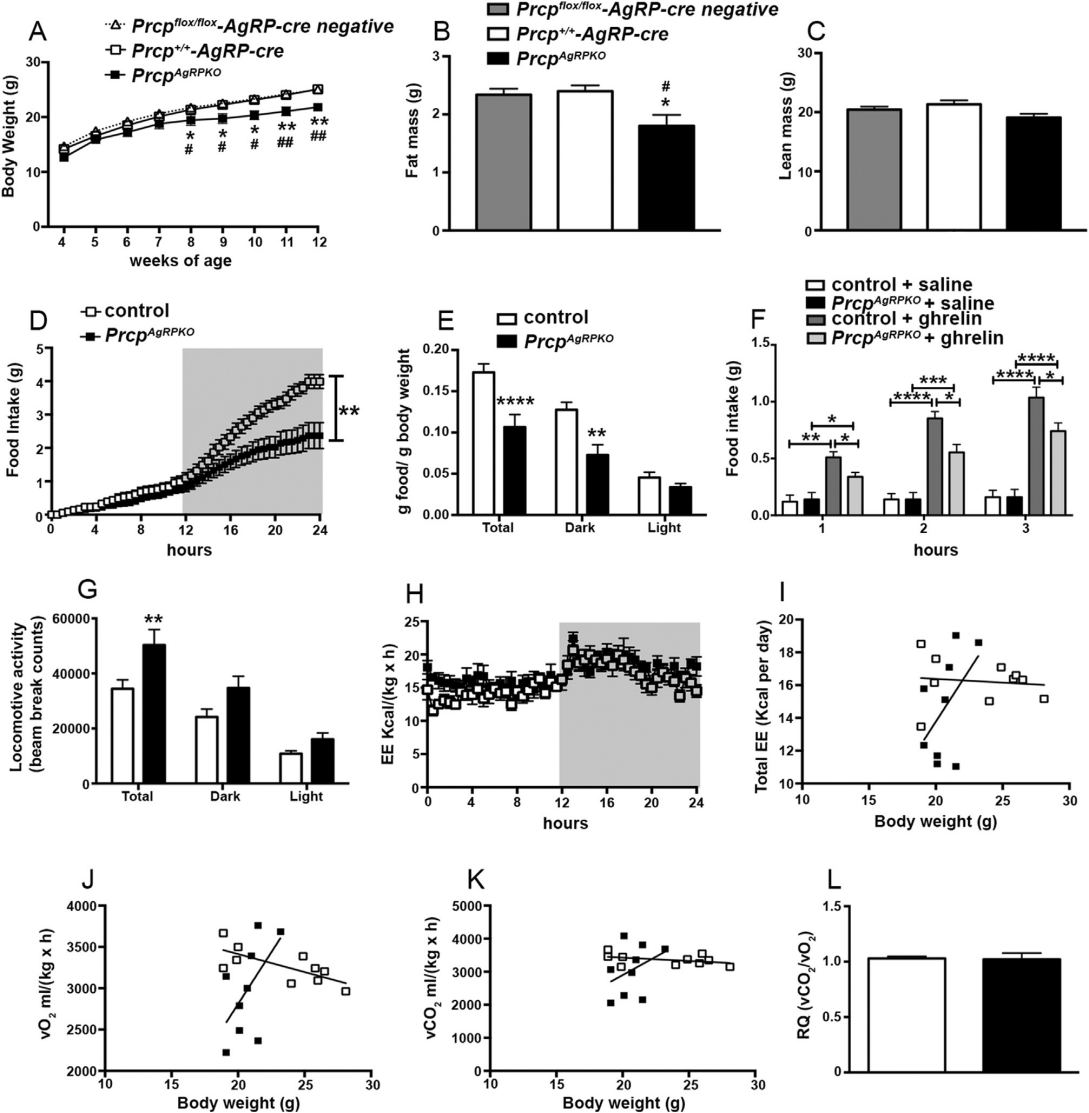
**Selective PRCP deletion in AgRP neurons affects metabolic phenotype in male mice.** (**A**–**C**) Graphs showing body weight (**A**), fat mass (**B**), and lean mass (**C**) of 3-month-old male *Prcp*^*flox/flox*^*-AgRP-cre negative* control mice (n = 5), *Prcp*^*+/+*^*-AgRP-cre* control mice (n = 10), and *Prcp*^*AgRPKO*^ male mice (n = 10). * = P < 0.05 compared to *Prcp*^*+/+*^*-AgRP-cre* mice; ** = P < 0.01 compared to *Prcp*^*+/+*^*-AgRP-cre* mice; # = P < 0.05 compared to *Prcp*^*flox/flox*^*-AgRP-cre* negative mice; ## = P < 0.01 compared to *Prcp*^*flox/flox*^*-AgRP-cre* negative mice. (**D** and **E**) Graphs showing food intake in control mice (n = 9) and *Prcp*^*AgRPKO*^ mice (n = 8). Results of food intake as total in the 24-h cycle and in the dark and light phases of the cycle. Gray area represents dark phases. (**F**) Graph showing food intake in male *Prcp*^*AgRPKO*^ and control mice (n = 4/5 per group) at 1 h, 2 h and 3 h after ip ghrelin administration. (**G**–**L**) Graphs showing locomotor activity (**G**), energy expenditure (**H** and **I**), O_2_ consumed (**J**), CO_2_ produced (**K**), and their ratio (**L**) of three months old male control (n = 9) and *Prcp*^*AgRPKO*^ mice (n = 8). All data are represented as mean ± SEM. * = P < 0.05; ** = P < 0.01; *** = P < 0.001; **** = P < 0.0001 compared to control mice.

To determine whether selective PRCP deletion in NPY/AgRP neurons affects metabolism, metabolic analyses were performed in *Prcp*^*AgRPKO*^ male mice. *Prcp*^*AgRPKO*^ male mice fed on a standard chow diet showed lower body weight (n = 25 per group; Figure 2A) starting at 8 weeks of age. The lower body weight was due to a significant reduction of fat mass (1.80 ± 0.60 g in *Prcp*^*AgRPKO*^ male mice; n = 9) compared to both male *Prcp*^*flox/flox*^-*AgRP-cre* negative (2.34 ± 0.31 g; n = 5) and *Prcp*^*+/+*^*-AgRP-cre* control mice (2.40 ± 0.22 g; n = 10; Figure 2B). No differences in lean mass were observed between the 3 experimental groups (20.44 ± 1.60 g in *Prcp*^*flox/flox*^-*AgRP-cre* negative male mice; 21.34 ± 1.50 g in *Prcp*^*+/+*^*-AgRP-cre* male mice; 19.09 ± 1.95 g in *Prcp*^*AgRPKO*^ male mice; Figure 2C). Since no differences were observed between *Prcp*^*flox/flox*^-*AgRP-cre* negative and *Prcp*^*+/+*^*-AgRP-cre* male mice, both groups were used in all experiments and indicated as control. The leaner phenotype was associated with decreased food intake (2.5 ± 0.42 g in male *Prcp*^*AgRPKO*^ mice and 4.02 ± 0.23 g in male controls; Figure 2D) specifically during the dark period (0.13 ± 0.03 g food/g BW in controls vs 0.07 ± 0.04 g food/g BW in *Prcp*^*AgRPKO*^ male mice; n = 9 and 8, respectively; Figure 2E). In addition, ghrelin-induced food intake at 1, 2, and 3 h after its ip administration was significantly lower in *Prcp*^*AgRPKO*^ male mice compared to male controls (Figure 2F). Furthermore, increases in locomotor activity (34418 ± 9700 beam break counts in controls vs 50333 ± 15853 beam break counts in *Prcp*^*AgRPKO*^ male mice; n = 9 and 8, respectively; Figure 2G) and energy expenditure (Figure 2H, I) were also observed in *Prcp*^*AgRPKO*^ male mice compared to male controls. Although vO_2_ and vCO_2_ were different between *Prcp*^*AgRPKO*^ mice and controls (Figure 2J, K), no change in respiratory quotient was observed (Figure 2J–L) between the experimental groups. Finally, analysis of body length showed a significant difference between the 2 experimental groups, with *Prcp*^*AgRPKO*^ male mice being shorter (9.28 ± 0.09 cm; n = 19) than male controls (9.69 ± 0.08 cm; n = 24; p = 0.001).

### 3.5 Selective PRCP deletion in AgRP neurons does not alter energy metabolism in female mice

Interestingly, no significant differences in body weight (Figure S3A), fat mass (2.34 ± 1.17 g in controls vs 2.21 ± 1.00 g in *Prcp*^*AgRPKO*^ female mice; n = 11 and 7 respectively; Figure S3B), and lean mass (15.51 ± 0.37 g in controls vs 13.95 ± 1.07 g in *Prcp*^*AgRPKO*^ female mice; n = 11 and 7 respectively; Figure S3C) were observed in female mice. However, similar to males, females *Prcp*^*AgRPKO*^ mice were significantly shorter (8.73 ± 0.08 cm; n = 15) than female controls (8.96 ± 0.07 cm; n = 20; p = 0.04). Analyses of food intake (Figure S3D and E), locomotor activity (Figure S3F), and energy expenditure (Figure S3G) showed no differences between female *Prcp*^*AgRPKO*^ (n = 7) and control mice (n = 6). However, when exposed to high fat diet (45% of fat), female *Prcp*^*AgRPKO*^ mice showed lower body weight gain compared to control female mice (Figure S3H; n = 8/9 per group). This difference was due to a significant reduction in fat mass (Figure S3I). No significant changes in lean mass were found (Figure S3J).

Altogether these data indicate that PRCP in AgRP neurons affects energy metabolism in female mice when challenged on HFD feeding.

### 3.6 Effects of PRCP deletion in AgRP neurons on circulating hormone levels in male mice

**Figure 3:**
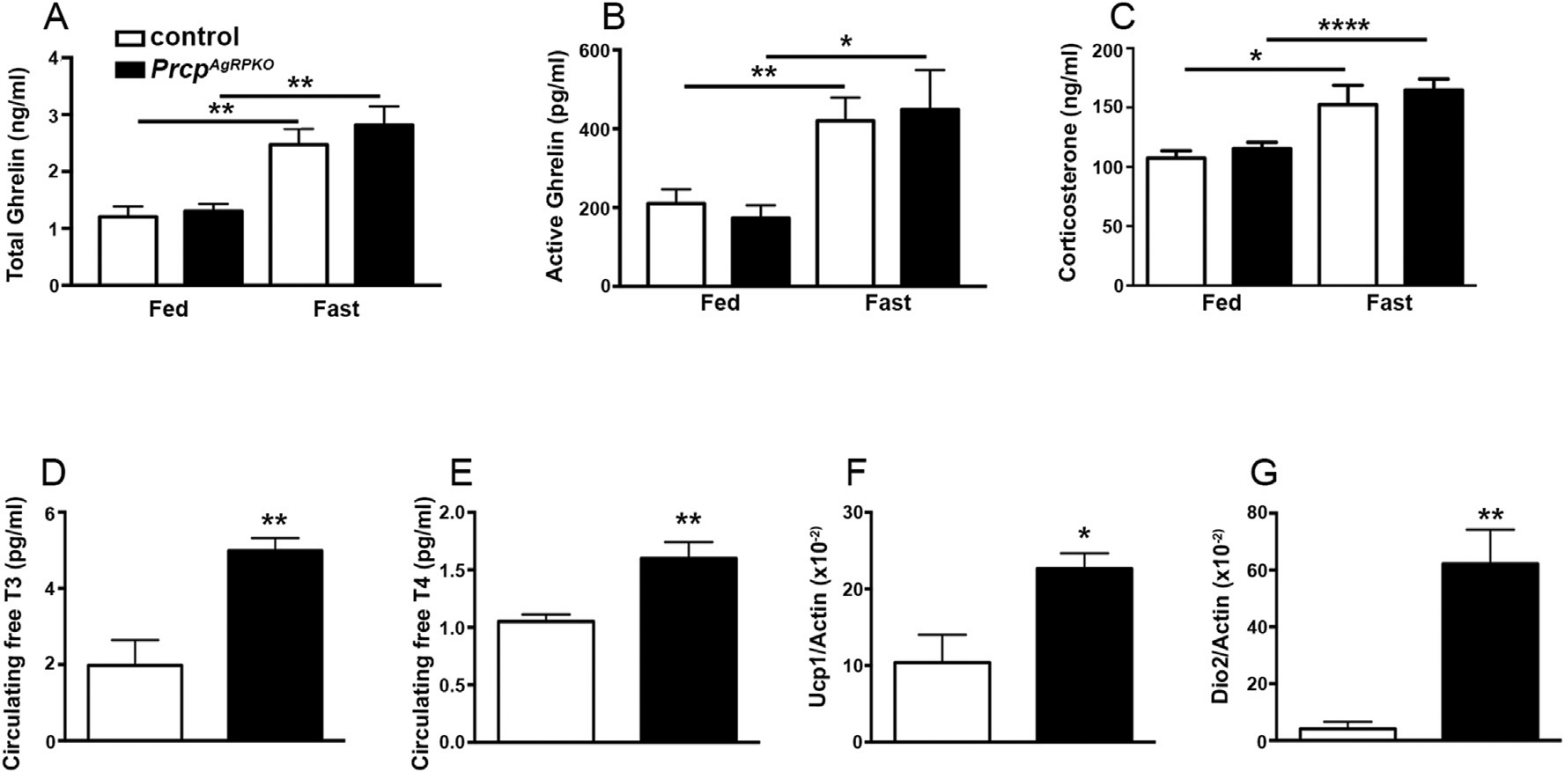
**Effects of PRCP deletion in AgRP neurons on circulating hormone levels in male mice.** (**A**) Graph showing serum total ghrelin levels (**A**) of 3-month-old male control (n = 5), and *Prcp*^*AgRPKO*^ mice (n = 5) on fed and fasted state. (**B**) Graph showing serum active ghrelin levels of male control (n = 10), and *Prcp*^*AgRPKO*^ mice (n = 5) on fed and fasted states. (**C**) Graph showing circulating corticosterone levels in male control (n = 5) and *Prcp*^*AgRPKO*^ mice (n = 8) on fed and fasted state. (**D** and **E**) Graphs showing the analysis of serum free T_3_ (**D**) and free T_4_ (**E**) levels in male control (n = 5), and *Prcp*^*AgRPKO*^ mice (n = 5) on fed state. (**F** and **G**) Graphs showing Real Time PCR data for Ucp1 (**F**) and Dio2 (**G**) in the brown adipose tissue of 3 months old male control and *Prcp*^*AgRPKO*^ mice (n = 5/4 per group). All data are represented as mean ± SEM. * = P < 0.05 compared to control mice; ** = P < 0.01 compared to control mice; **** = P < 0.0001 compared to control mice.

Analyses of metabolic hormones showed no differences in the levels of total (n = 5 per group; Figure 3A) and active ghrelin (n = 5 per group; Figure 3B) and corticosterone (n = 5 per group; Figure 3C) in fed or fasted states between male *Prcp*^*AgRPKO*^ and control mice. However, fasting levels of T_3_ (1.98 ± 0.67 pg/ml in male controls vs 4.99 ± 0.34 pg/ml in male *Prcp*^*AgRPKO*^ mice; n = 4 and 5 respectively; Figure 3D) and T_4_ (1.05 ± 0.06 pg/ml in male controls vs 1.60 ± 1.14 pg/ml in male*Prcp*^*AgRPKO*^ mice; n = 5 and 3 respectively; Figure 3E) were significant elevated in male *Prcp*^*AgRPKO*^ mice compared to male controls. Furthermore, significant increases in markers of BAT thermogenesis such as uncoupling protein 1 (Ucp1; n = 4 per group; Figure 3F) and deiodinase type 2 (Dio2; n = 4 per group; Figure 3G) were also observed in male *Prcp*^*AgRPKO*^ mice compared to controls.

### 3.7 Selective PRCP deletion in AgRP neurons improves glucose metabolism in male mice

**Figure 4:**
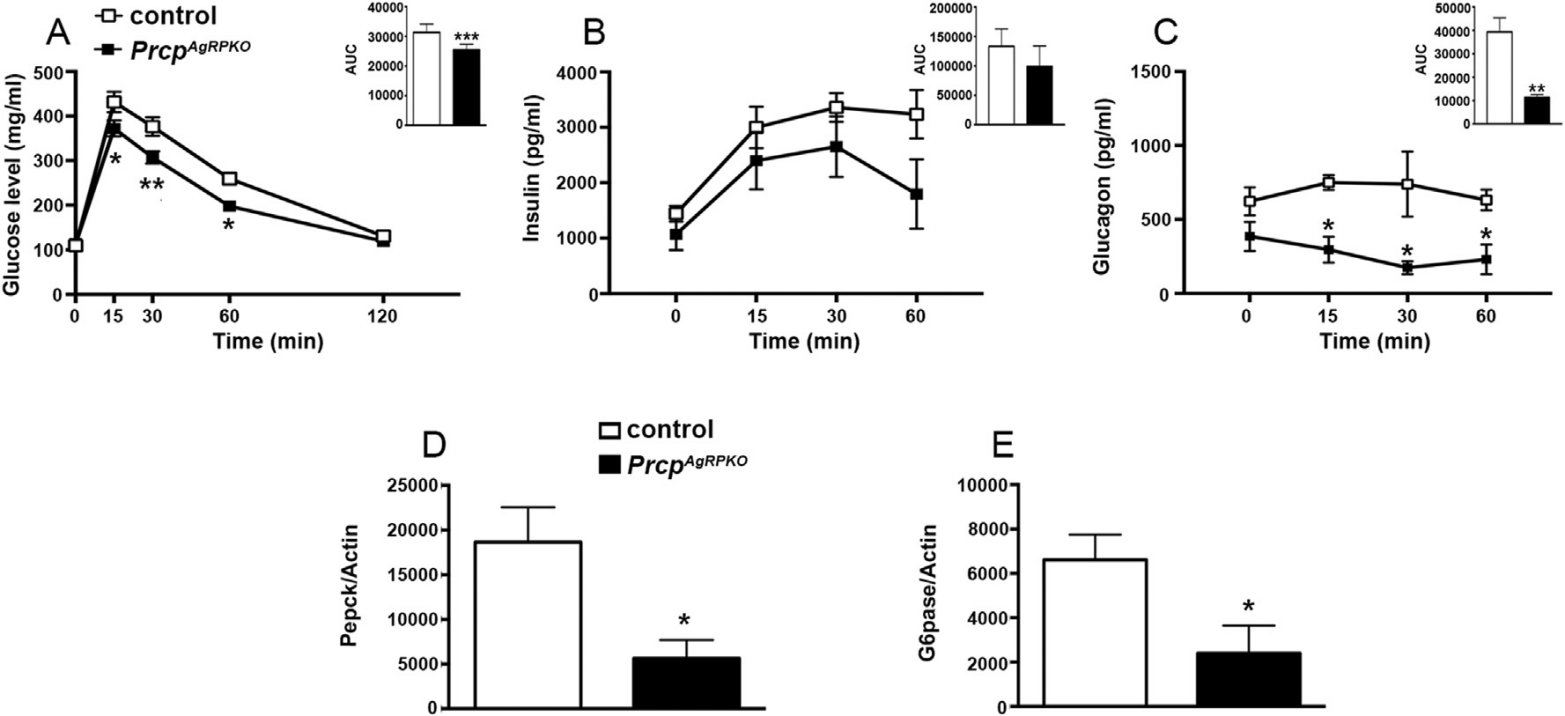
**Selective PRCP deletion in AgRP neurons improves glucose metabolism in male mice.** (**A**) Graph showing glucose tolerance test in 3-month-old male control and *Prcp*^*AgRPKO*^ mice (n = 10 per group). (**B** and **C**) Graphs showing circulating insulin (**B**) and glucagon (**C**) levels during glucose tolerance test in control male control (n = 5) and *Prcp*^*AgRPKO*^ mice (n = 5). (**D** and **E**) Graphs showing mRNA levels of liver Pepck (**D**) and G6Pase (**E**) in male control (n = 5) and *Prcp*^*AgRPKO*^ mice (n = 5) in fed state. All data are represented as mean ± SEM. * = P < 0.05 compared to control mice; ** = P < 0.01 compared to control mice.

In agreement with the observed lean phenotype, three months old *Prcp*^*AgRPKO*^ male mice showed improved glucose tolerance (n = 10 per group; Figure 4A) that was associated with no significant changes in insulin levels (n = 5 per group; Figure 4B) but reduced circulating glucagon levels (n = 4 per group; Figure 4C) and decreased hepatic mRNA levels for gluconeogenesis enzymes such as PEPCK (n = 4 per group; Figure 4D) and G6Pase (n = 4 per group; Figure 4E).

No differences in glucose metabolism were observed in female *Prcp*^*AgRPKO*^ mice compared to controls (data not shown).

### 3.8 Selective PRCP deletion in AgRP neurons increases hypothalamic α-MSH levels

**Figure 5:**
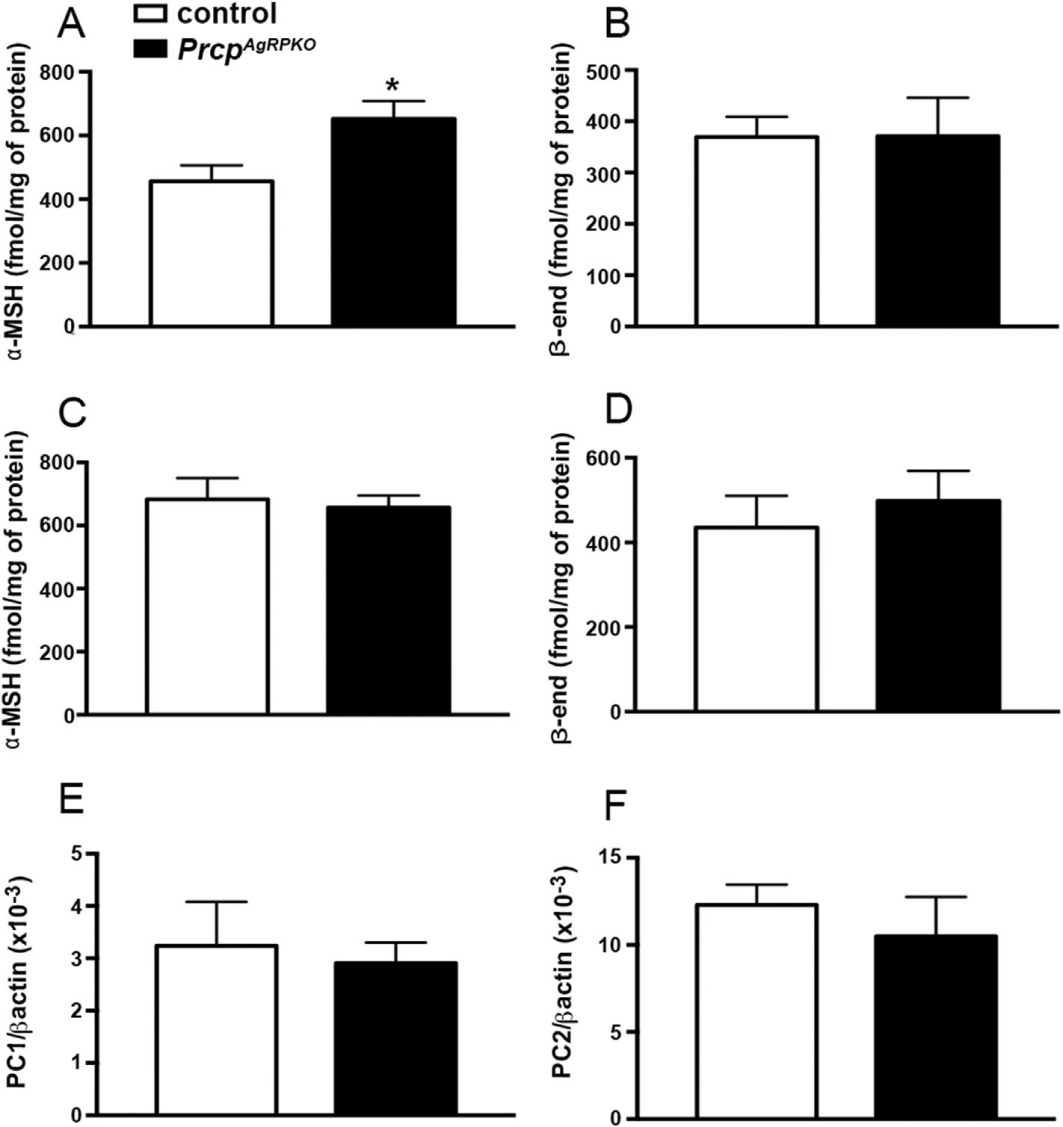
**α-MSH and â-endorphin levels in *Prcp*^*AgRPKO*^ mice.** (**A**) Graph showing the α-MSH levels (expressed as fmol α-MSH/mg protein) in the hypothalamus of male *Prcp*^*AgRPKO*^ mice (n = 9) compared to control mice (n = 9). (**B**) Graph showing the β-endorphin levels in the hypothalamus of male *Prcp*^*AgRPKO*^ mice (n = 8) compared to controls (n = 9). (**C** and **D**) Graphs showing α-MSH and β-endorphin levels in the hypothalamus of female *Prcp*^*AgRPKO*^ (n = 6) compared to controls mice (n = 6). (**E** and **F**) Graphs showing PC1 (E) and PC2 (F) mRNA levels in the hypothalamus of male *Prcp*^*AgRPKO*^ (n = 5) compared to control mice (n = 5). Data represent the mean ± SEM. * = P < 0.05 compared to control mice.

PRCP inactivates hypothalamic α-MSH levels [12]. To assess whether selective deletion of PRCP in AgRP neurons alters hypothalamic α-MSH levels, we measured α-MSH levels in hypothalami of 3-months old *Prcp*^*AgRPKO*^ and control mice. A significant increase in α-MSH levels was found in male *Prcp*^*AgRPKO*^ mice (652.5 ± 55.91 fmol/mg protein; n = 9) compared to controls (456.2 ± 49.78 fmol/mg protein; n = 9; Figure 5A). On the other hand, no change in β-endorphin, also a POMC-derived peptide, was observed between the 2 experimental male groups (369.5 ± 39.54 fmol/mg protein in controls vs 371.2 ± 74.65 fmol/mg protein in *Prcp*^*AgRPKO*^ mice; n = 9 per male controls and n = 7 per *Prcp*^*AgRPKO*^ male mice; Figure 5B), suggesting a role for PRCP in AgRP neurons in altering selectively α-MSH. Interestingly, no differences in either hypothalamic α-MSH (682.7 ± 67.41 fmol/mg protein in female controls vs 657.1 ± 38.05 fmol/mg protein in female *Prcp*^*AgRPKO*^ mice; n = 6 per group; Figure 5C) or β-endorphin levels (435.1 ± 75.03 fmol/mg protein in female controls vs 497.9 ± 70.82 fmol/mg protein in female *Prcp*^*AgRPKO*^ mice; n = 5 per female controls and n = 6 per female *Prcp*^*AgRPKO*^ mice; Figure 5D) were found between *Prcp*^*AgRPKO*^ and control female mice.

In further support of the role of PRCP in the inactivation of α-MSH, we next measured the expression of enzymes, such as pro-hormone convertase 1 and 2 (PC1 and PC2), involved in the production of POMC-derived peptides. No difference in their expression levels was observed between control and *Prcp*^*AgRPKO*^ male mice (Figure 5E, F).

Thus, these data indicate that deletion of PRCP from AgRP neurons selectively alters α-MSH levels and in a sex-specific manner.

### 3.9 Selective PRCP deletion in AgRP neurons increases neuronal activation in the paraventricular nucleus of the hypothalamus

**Figure 6:**
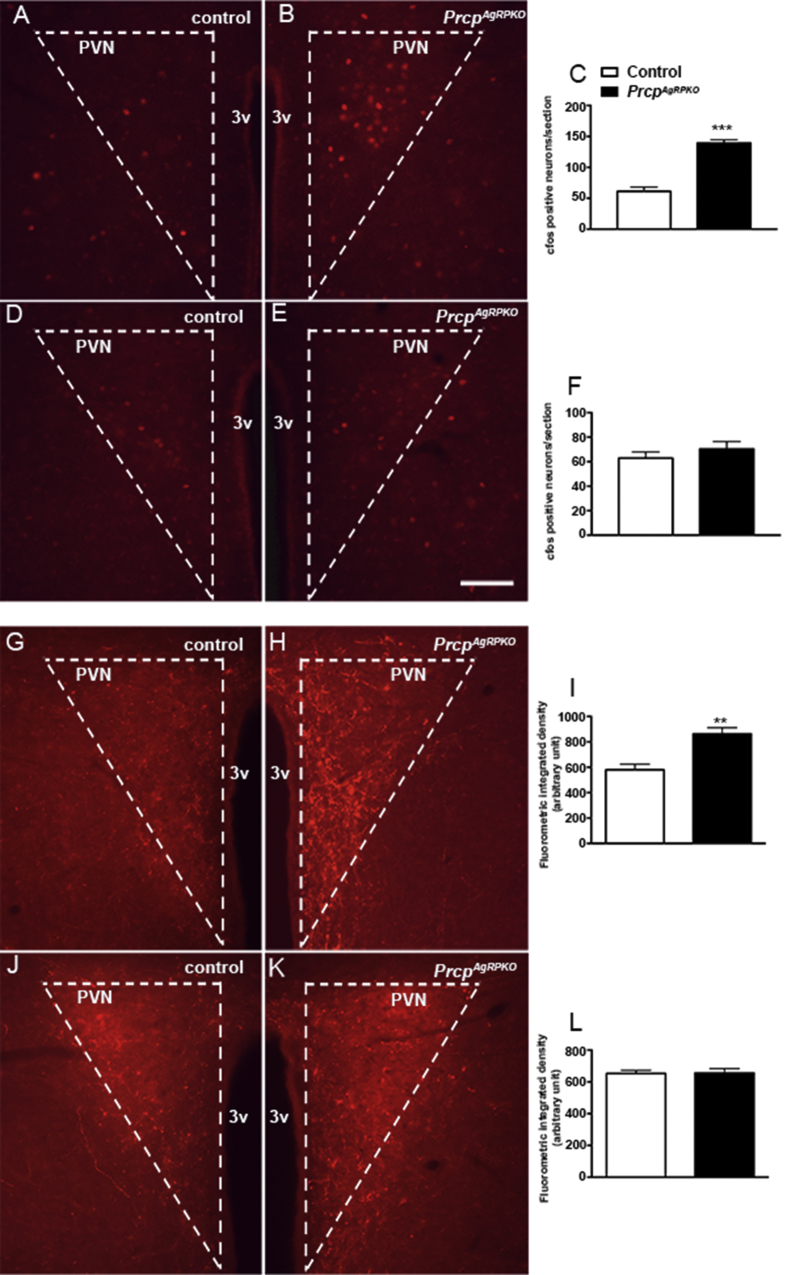
**Role of PRCP deletion in AgRP neurons in the paraventricular nucleus of the hypothalamus.** (**A** and **B**) Representative hypothalamic sections from a control male mouse (**A**) and a *Prcp*^*AgRPKO*^ male mouse (**B**) fasted overnight and immunostained for cfos (red) in the hypothalamic paraventricular nucleus (PVN). (**C**) Quantification of cfos expression in hypothalamic paraventricular nucleus neurons of fasted control male mice (n = 3) and fasted *Prcp*^*AgRPKO*^ male mice (n = 3). (**D** and **E**) Representative micrographs of hypothalamic sections showing immunostaining for cfos from a fasted control female mouse (n = 3) and a fasted *Prcp*^*AgRPKO*^ female mouse (n = 3) in the PVN. (**F**) Graph showing the quantification in hypothalamic paraventricular nucleus neurons immunostained for cfos in fasted control female mice (n = 3) and fasted *Prcp*^*AgRPKO*^ female mice (n = 3). (**G** and **H**) Representative light micrographs showing immunostained á-MSH fibers in the PVN of a fasted control male mouse (n = 4) and a fasted *Prcp*^*AgRPKO*^ male mouse (n = 4). (**I**) Graph showing the integrated density quantification of á-MSH fibers in the PVN of fasted control and *Prcp*^*AgRPKO*^ male mice (n = 4 per group). (**J** and **K**) Representative micrographs of hypothalamic sections from a fasted control female mouse (n = 4) and a fasted *Prcp*^*AgRPKO*^ female mouse (n = 4) immunostained for á-MSH fibers in the PVN. (**L**) Graph showing the quantification of the fluorescent density of á-MSH fibers in the PVN of fasted control female mice (n = 4) and fasted *Prcp*^*AgRPKO*^ female mice (n = 4). 3v = third ventricle; PVN = paraventricular nucleus of the hypothalamus. Bar scale in **E** (for **A**, **B**, **D**, **G**, **H**, **J** and **K**) represents 100 ìm. All data are represented as mean ± SEM. ** = P < 0.01compared to control mice; *** = P < 0.001 compared to control mice.

To determine whether PRCP deletion in AgRP neurons alters the activation of PVN neurons, a target of the NPY/AgRP neurons, we analyzed cfos immunoreactivity in the PVN of 3-months old fasted *Prcp*^*AgRPKO*^ male and female mice and their controls. A greater increase of cfos positive cells was detected in the PVN of male *Prcp*^*AgRPKO*^ mice (139.3 ± 5.21 cfos positive cells/section; n = 3 mice; Figure 6A–C) compared to controls (60.47 ± 6.87 cfos positive cells/section; n = 3; Figure 6A, C). However, and in agreement with the α-MSH levels in female mice, no changes in cfos immunoreactivity was observed in the PVN of female *Prcp*^*AgRPKO*^ mice and their controls (64.0 ± 8.51 cfos positive cells/section in controls vs 70.67 ± 61.12 cfos positive cells/section in *Prcp*^*AgRPKO*^ female mice; n = 3 per group; Figure 6D–F).

### 3.10 Selective PRCP deletion in AgRP neurons increases α-MSH immunoreactivity in the paraventricular nucleus of the hypothalamus

To determine whether PRCP deletion in AgRP neurons alters α-MSH projections to the PVN, we then analyzed α-MSH immunoreactivity in the PVN of *Prcp*^*AgRPKO*^ male and female mice and their controls. An increase in α-MSH fiber staining was observed in the PVN of male *Prcp*^*AgRPKO*^ mice compared to male controls (Figure 6G–I). No difference in PVN α-MSH immunoreactivity was detected between female *Prcp*^*AgRPKO*^ mice and their controls (Figure 6J–L).

### 3.11 Selective PRCP deletion in AgRP neurons affects feeding via the PVN melanocortin receptors

**Figure 7:**
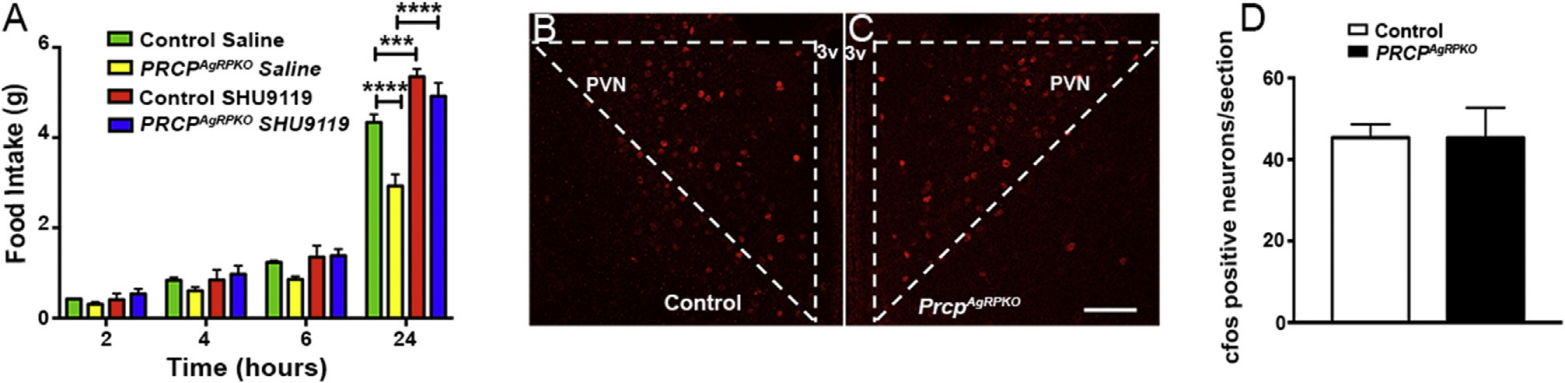
**Selective SHU9119 administration in the PVN increases feeding and neuronal activation in male *Prcp*^*AgRPKO*^ mice.** (**A**) Graph showing feeding response after bilateral PVN injections of SHU9119 in 3-month-old male control and *Prcp*^*AgRPKO*^ mice (n = 5 per group). (**B** and **C**) Representative hypothalamic sections from a control male mouse (**B**) and a *Prcp*^*AgRPKO*^ male mouse (**C**) injected with SHU9119 immunostained for cfos (red) in the hypothalamic paraventricular nucleus (PVN). (**D**) Quantification of cfos expression in hypothalamic paraventricular nucleus neurons of control male mice (n = 5) and *Prcp*^*AgRPKO*^ male mice (n = 5) injected with SHU9119. 3v = third ventricle; PVN = paraventricular nucleus of the hypothalamus. Bar scale in **C** (for **B**) represents 100 ìm. All data are represented as mean ± SEM. *** = P < 0.001; **** = P < 0.0001.

To further test the hypothesis that the deletion of PRCP from AgRP neurons affects metabolism through the PVN melanocortin receptor-expressing neurons, we administered SHU9119, a potent melanocortin receptors antagonist, and vehicle in the PVN of male control and *Prcp*^*AgRPKO*^ mice and measured food intake. Twenty-four hours food intake showed that SHU9119-injected male *Prcp*^*AgRPKO*^ mice ate significantly more than vehicle-injected *Prcp*^*AgRPKO*^ male mice (Figure 7A), reaching an intake similar to that of SHU9119-injected control male mice. This result suggests that the effect of PRCP deletion in AgRP neurons on feeding is mediated by the PVN melanocortin receptor-expressing neurons, thus supporting the hypothesis that PRCP released at the synaptic level, by degrading α-MSH, regulates melanocortin signaling. Furthermore, analysis of cfos immunoreactivity in the PVN of male control and *Prcp*^*AgRPKO*^ mice injected with SHU9119 showed no differences between the 2 experimental groups (Figure 7B–D).

## 4 Discussion

The unidirectional synaptic interaction between the NPY/AgRP and POMC neurons [30] together with the antagonistic action of AgRP on α-MSH binding to MC4R-expressing target neurons provides a basal blueprint for metabolism regulation by the hypothalamic melanocortin system. Our current study provides evidence that PRCP expressed in NPY/AgRP neurons represents an additional regulatory mechanism in this system in the regulation of food intake and energy metabolism. We have shown that PRCP, an enzyme involved in the degradation of α-MSH [12], is expressed in NPY/AgRP neurons. We found that the majority of NPY/AgRP neurons express PRCP, suggesting that these neurons are in an ideal position to determine the efficacy of the released α-MSH via PRCP release. To assess whether PRCP is released, we measured PRCP in the medium of hypothalamic explants after stimulation by ghrelin, which promotes PRCP expression [29] and activates NPY/AgRP neurons [2]. Compared to the basal glucose treatment, ghrelin treatment induced a significant increase in PRCP levels in the medium, suggesting that activation of neurons responsive to ghrelin can induce the release of PRCP. Furthermore, we also found the high glucose levels significantly decreased PRCP secretion in the medium, thus indicating that glucose, by inhibiting neuronal activation, affects PRCP release. These data, together with previous published studies [14], [15], [16], suggest that PRCP is secreted at the synaptic levels. In addition, because NPY/AgRP neurons are activated by ghrelin and inhibited by glucose, these data also suggest that NPY/AgRP neurons expressing PRCP can release the enzyme at the synaptic level where it inactivates α-MSH to modulate melanocortin signaling.

We then generated mice with a selective deletion of PRCP in AgRP neurons and showed that they have increased hypothalamic α-MSH levels and reduced ghrelin-induced PRCP secretion in hypothalamic explants compared to control mice. In agreement with these, *PRCP*^*AgRPKO*^ mice showed decreased food intake and increased energy expenditure and locomotor activity, that translated in a leaner phenotype in male but not female mice. This sex difference in PRCP's impact on metabolism is in line with a study showing significant associations between the metabolic syndrome and polymorphisms on the PRCP gene only in men but not in women [31]. However, when female mice were challenged with a high fat diet (45%), female *Prcp*^*AgRPKO*^ mice were protected from diet-induced obesity.

Similar to *Prcp*^*AgRPKO*^ mice generated and analyzed in this study, mice hypomorph for PRCP also were shown to have decreased body weight with reduced fat mass and feeding compared wild type controls [12], suggesting that their leaner phenotype could be mediated by the hypothalamic NPY/AgRP neurons.

Altogether, our observations suggest that PRCP, released at the synaptic level by cells, including the NPY/AgRP, acts by inactivating α-MSH released by POMC neurons at the same target areas. To test for that, selective blockade of MC4R by SHU9119 in the paraventricular nucleus of the hypothalamus, a major target area of both AgRP and POMC fibers, was performed in control and *Prcp*^*AgRPKO*^ mice and food intake examined. As expected, SHU9119 significantly increased food intake in control mice compared to the vehicle-injected controls. When SHU9119 was injected in the PVN of male *Prcp*^*AgRPKO*^ mice, food intake was significantly increased compared to vehicle-injected *Prcp*^*AgRPKO*^ mice, reaching a feeding level that was not different from that observed in SHU9119-injected control mice. Consistent with the feeding measurements, PVN neuronal activation was not different between SHU9119-injected *Prcp*^*AgRPKO*^ and SHU9119-injected control mice. This suggests that PRCP in NPY/AgRP neurons affects food intake via modulation of the PVN melanocortin receptors.

Interesting, the stimulation of food intake after SHU9119 injections in both control and *Prcp*^*AgRPKO*^ male mice was not observed in the first hours from its administration but was evident after 24 h from its injection. This effect could be attributed to the time of SHU9119 administration and the metabolic status of the mice. Indeed, similar to our experimental paradigm, Fan and collaborators [32] observed that daytime food intake in animals fed ad libitum was not stimulated by intra-cerebroventricular administration of SHU9119. They observed that SHU9119 stimulated food intake only when administered to animals before lights out. Furthermore, SHU9119 induced a significant increase in daytime food intake only when mice were overnight food deprived [32].

Key aspects of the melanocortin system are the innervation of POMC neurons by NPY/AgRP fibers derived from adjacent arcuate NPY/AgRP neurons [30] and the efferents of both of these neuronal populations to CNS, such as the hypothalamic PVN that expresses MC4Rs [2]. Our study argues for an additional regulatory component of the melanocortin signaling. PRCP released from AgRP fibers inactivates α-MSH at the synaptic level further contribute to the orexigenic tone of the AgRP system. In further support of the role of PRCP in the regulation of the melanocortin system, *PRCP*^*AgRPKO*^ mice showed a significant decrease in body length compared to controls. Besides α-MSH, PRCP in AgRP neurons could also act on other, yet unidentified peptides having proline as the penultime amino acid, the function of which also could be related to metabolism and the melanocortin system.

## 5 Conclusion

In summary, our data provide evidence that PRCP in NPY/AgRP neurons modulate melanocortin signaling in the hypothalamic paraventricular nucleus via control of α-MSH degradation. This mechanism is sex-specific and represents an additional process through which the orexigenic NPY/AgRP neurons regulate the output of the anorexigenic POMC cells.

## Author contributions

GB, SJ and JDK conducted experiments and acquired and analyzed data. SD designed the research studies, analyzed data, and wrote the manuscript.
