# Supplementary material for: Prolyl carboxypeptidase in Agouti-related Peptide neurons modulates food intake and body weight
Source: Mol Metab. 2018 Feb 8;10:28–38. doi: 10.1016/j.molmet.2018.02.003 (PMC5985234; doi:10.1016/j.molmet.2018.02.003)
Supplement: Supplementary file 1 [file mmc1.docx]

**
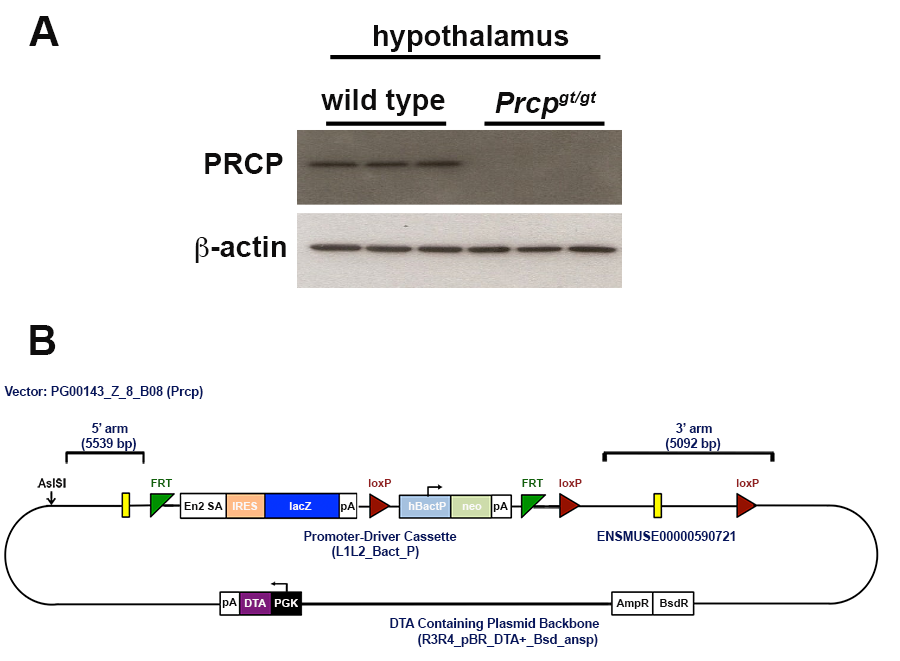
Figure S1: Antisera validation and Generation of mice used in this study**

(**A**) Western blot analysis of PRCP and β-actin in the hypothalamus of wild type and whole body PRCP gene trap mice (*Prcp^gt/gt^*) [12].

(**B**) Schematic diagram showing *Prcp^flox/flox^* constructs.


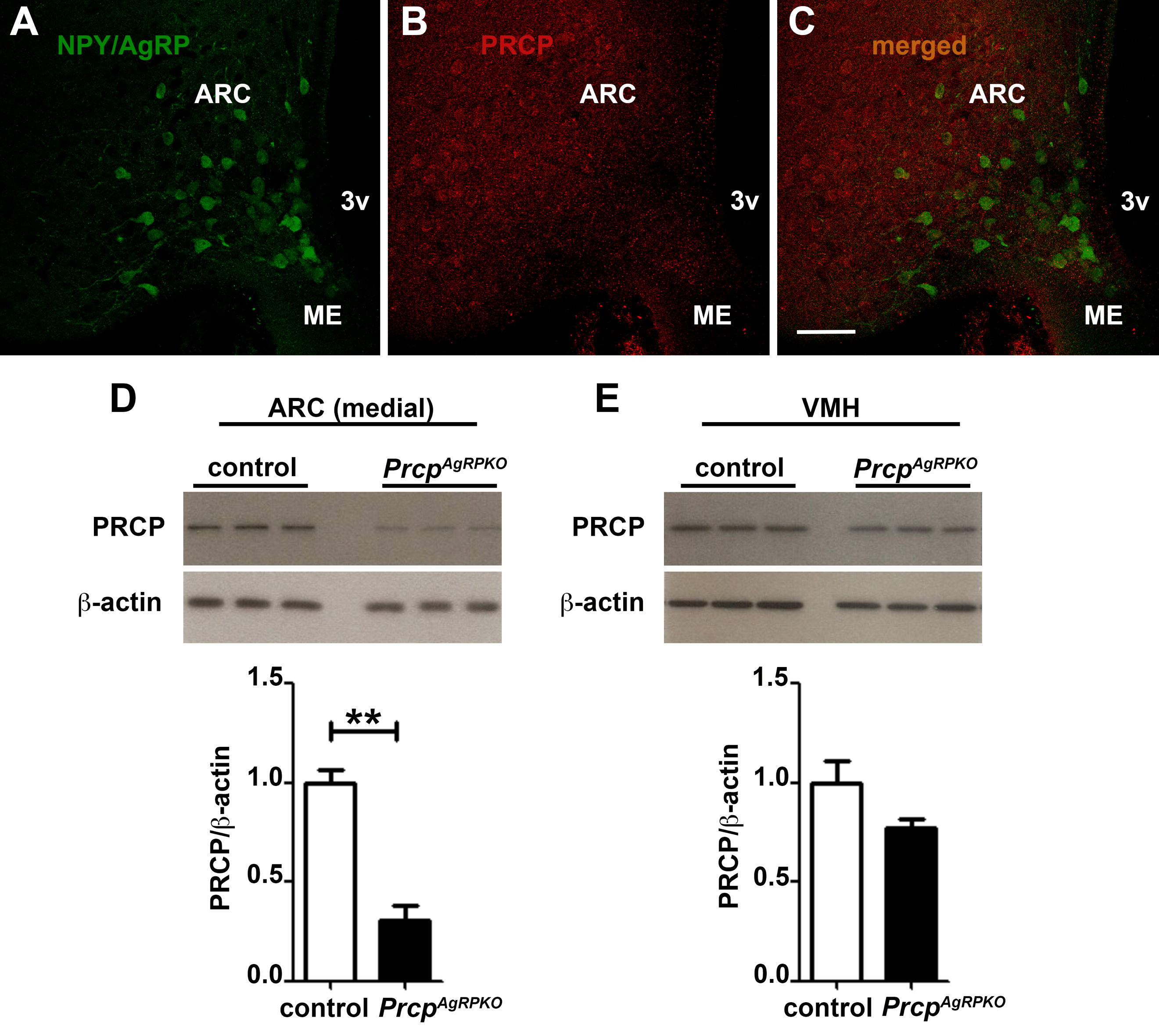


**Figure S2: PRCP expression in the NPY/AgRP neurons of *Prcp^AgRPKO^* mice**

(**A**-**C**) Representative light micrograph showing immunostaining for GFP (green; **A**), PRCP (red; **B**) and merged (**C**) in the ARC of the hypothalamus of *Prcp^AgRPKO^* mouse.

3v= third ventricle; ARC= arcuate nucleus; ME= median eminence. Bar scale in **C** (for all panels) represents 50um.

(**D** and **E**) Western blot analysis and quantification of PRCP protein levels in the arcuate nucleus (medial portion) and the ventromedial nucleus (VMH) of *Prcp^AgRPKO^* and control mice (n=3/group). **=P<0.01


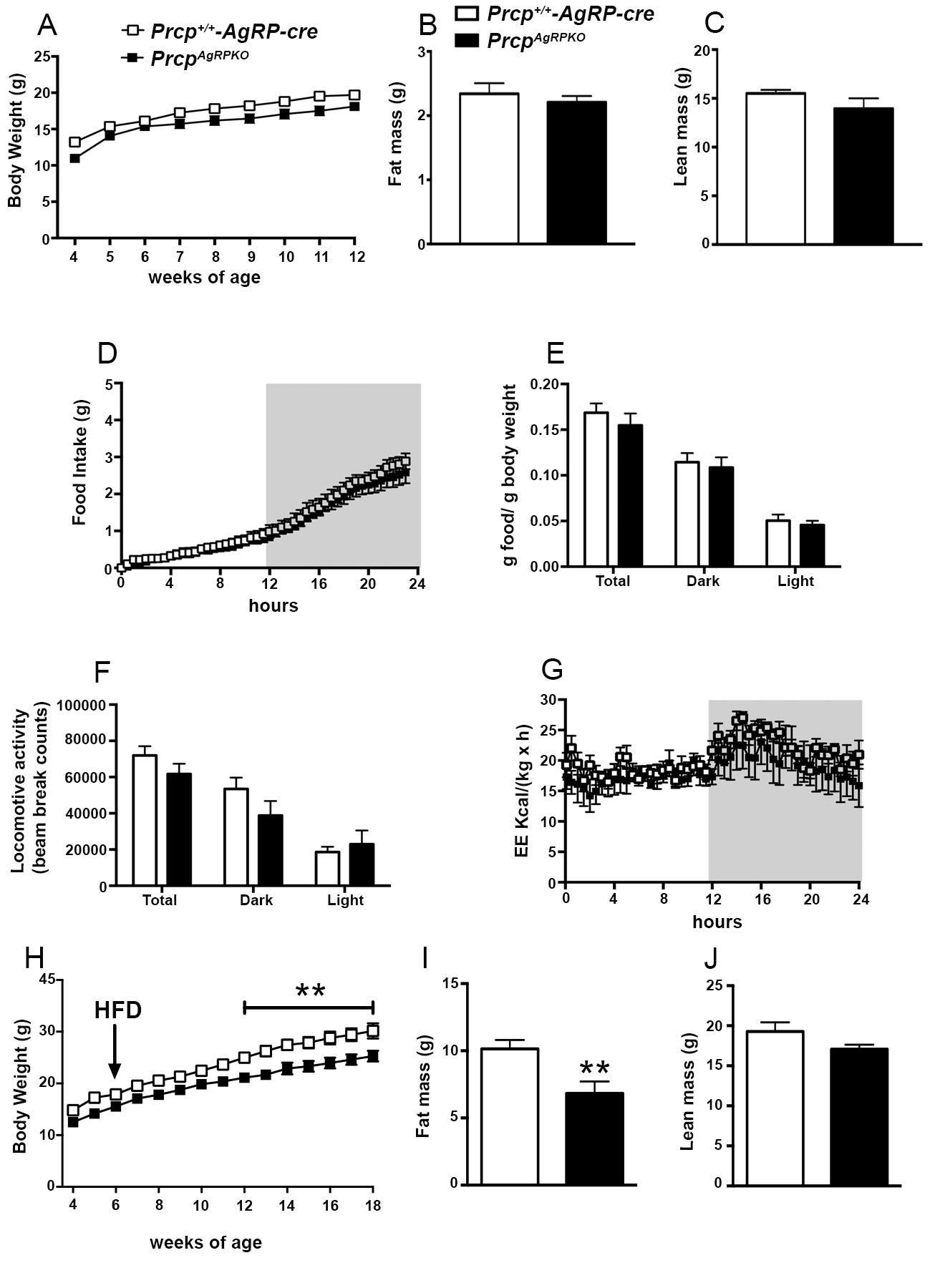


**Figure S3: Selective PRCP deletion in AgRP neurons does not affects metabolic phenotype in female mice**

(**A**-**C**) Graphs showing body weight (**A**), fat mass (**B**) and lean mass (**C**) of 3 months old female

*Prcp^+/+^-AgRP-cre* (*n*=12) and *Prcp^AgRPKO^* mice (*n*=8).

(**D** and **E**) Graphs showing food intake in control female *Prcp^+/+^-AgRP-cre* (*n* =6), and *Prcp^AgRPKO^* mice (*n*=7). Results of food intake as total in the 24-h cycle and in the dark and light phases of the cycle. Gray area represents dark phases.

(**F** and **G**) Graphs showing locomotor activity (**F**) and energy expenditure (**G**) of 3 months old female *Prcp^+/+^-AgRP-cre* (*n*=6) and *Prcp^AgRPKO^* (*n*=8).

(**H**-**J**) Graphs showing body weight (**H**), fat mass (**I**) and lean mass (**J**) of female *Prcp^+/+^-AgRP-cre* (*n*=9) and *Prcp^AgRPKO^* mice (*n*=8) exposed to HFD starting at 6 weeks of age.

All data are represented as mean ± SEM. **=P<0.01
